# Application of non-uniform film thickness concept in predicting deviated gas wells liquid loading

**DOI:** 10.1016/j.mex.2019.10.002

**Published:** 2019-10-11

**Authors:** Emeka Emmanuel Okoro, Cynthia I. Bassey, Samuel E. Sanni, Muhammad Gul Bahar Ashiq, Angela O. Mamudu

**Affiliations:** a Petroleum Engineering Department, Covenant University Ota, Nigeria; b Chemical Engineering Department, Covenant University Ota, Nigeria; c Physics Department, College of Science, Basic and Applied Scientific Research Center, Imam Abulrahman Bin Faisal University, Dammam, Saudi Arabia

**Keywords:** Liquid film reversal and uniform film concepts. Application of the concept of non-uniform film thickness in predicting liquid loading in deviated gas wells, Liquid loading, Non-uniform film thickness, Model, Gas well

## Abstract

Liquid loading causes undesirable occurrences such as premature death of wells, as well as significant reduction in production. However, most available models consider vertical wells and only a few focus on deviated gas wells. In order to reduce the impact of liquid loading on gas production, gas well load-up should be diagnosed at its early stage so as to proffer adequate solution. Unfortunately, most gas wells will experience liquid loading at some stage or point in their production life. Therefore, it is of utmost importance to predict liquid loading at the early life of such wells in order to develop apt liquid management strategies as corrective measures. Liquid film flow reversal concept has been identified as one of the major concepts responsible for the occurrence of liquid loading in deviated gas wells. This study develops an improvement on Chen’s liquid loading model. The model specifically introduces the concept of non-uniform film thickness around the pipe wall, as against previous works which considered uniform film thickness. A modified friction factor is also introduced to account for large film thicknesses around the pipe wall. Results from the model were compared with those of previous models, and data from published literature was used to validate the new model. The new model gave accurate predictions for 11 of 12 unloaded wells while for the loaded wells, the estimated data gave accuracies for 29 out of 30 loaded wells. This then implies that the new model is accurate for predicting liquid loading in deviated gas wells.

• Predictions from the new model show a good improvement over existing models.
• The uniform film assumption made in Chen liquid loading model was modified, and a different interfacial friction factor was applied.
• The method proposed in this study introduces the concept of non-uniform film thickness around the pipe wall as against previous works which considered uniform film thickness.

## Graphical abstract

**Figure:**
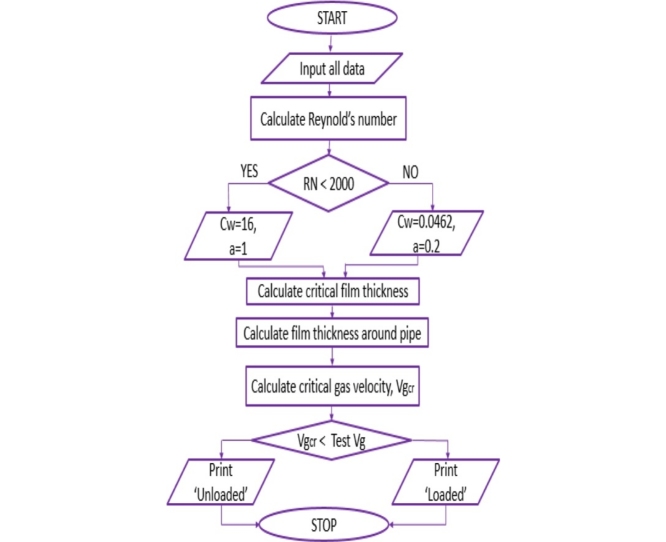

**Specification Table**

**Table:** 

Subject area:	Energy
More specific subject area:	Liquid loading in deviated gas wells
Method name:	Liquid film reversal and uniform film concepts. Application of the concept of non-uniform film thickness in predicting liquid loading in deviated gas wells
Name and Reference of original method:	Chen, D., Meng, H., Yao, Y., Fu, G., and Xie, S., A new model for predicting liquid loading in deviated gas wells. *Journal of Natural Gas Science and Engineering.* Volume 34, pp. 178–184, 2016.
Resource availability:	N/A

## Method details

### Overview

There is an ever-rising demand for natural gas for the major reason that it is a cleaner source of energy compared to oil and coal. Natural gas is not only cleaner than oil, but higher recoveries i.e. above 90% is more feasible with gas relative to oil. To meet the increasing demand for natural gas, it is of great essence to produce a well at an optimized rate over a long time frame. Also, improvement of recovery efficiencies from already existing natural gas wells is encouraged. However, there exists a major operational constraint in natural gas production, identified as “liquid loading”. Basically, liquid loading depicts the failure of gas to successfully transport co-produced liquids from the wellbore as the gas is being produced. Mist flow exists at high gas velocities in wells where the liquid are dispersed in the gas. Some liquids form a thin film on the wall of the wellbore while others are entrained within the gas. A low-pressure gradient exists in the well when the gas to liquid volume ration is relatively large. Turner et al. [1] developed a model based on the theory of critical gas velocity, which was used to predict the onset of liquid loading in a typical well.

Nosseir et al. [2] used Turner’s correlation as their basis for the discovery of a new approach that predicts liquid loading. They considered the influence of Reynolds number on drag force and found that Turner’s correlation estimates erroneous drag force values, thus resulting in the need for an upward adjustment of 20% in the estimated liquid loadings. Veeken et al. [3] defined the concept of Turner’s ratio as the ratio of the actual and minimum flow rates for continuous liquid removal from a producing well. They considered both vertical and deviated wells, and obtained similar critical flow rates for both wells. Guo et al. [4] accounted for the minimum kinetic energy of gas flow in their modified version of the Turner et al.’s equation. Belfroid et al. [5] also modified Turner’s equation and suggested that inclination angle, tubing outflow, flow regime, and reservoir inflow relations should be considered when predicting the critical flow rate of a gas stream.

In the oil and gas industry, “critical flow rate evaluation” has been the most widely used and generally accepted approach for predicting the genesis of liquid loading, as critical gas velocity (gas flow rate) is believed to be the major dominating factor that prevents liquid loading in gas wells [[6], [7], [8], [9]]. Fadairo et al. [10] observed in their study that, the aforementioned Guo et al. model, underestimates the critical flow rate in a gas well, however, they used an iterative method to obtain the critical flow rate. Luo [11] developed a new model to predict liquid loading by considering non-uniform film thickness around a tubular pipe. They observed that at an angle of 30 degrees, the largest film thickness is experienced. They compared their models with the entrained droplet model and came to the conclusion that the film flow model was more accurate and hence, is a preferred theory as the reason behind the genesis of liquid loading in gas wells [13]. The “entrained drop movement model” suggests that the onset of liquid loading can be attributed to the falling of liquid particles/droplets entrained in the high velocity gas core [12,15,16]. This is because the model views critical velocity as a function of gravitational, buoyancy and drag forces.

In lieu of these efforts, it was observed that previous studies established their findings on the Turner et al. model which assumes that the gas well flows in a dispersed phase. Nevertheless, recent research works have deviated from the popular belief that liquid loading is initiated when liquid droplets fall. Zhou and Yuan [14] highlighted that apart from gas velocity, the amount of liquid present in gas flow could also be a main factor for experiencing liquid loading. Luo [18] developed a new model to predict liquid loading by considering non-uniform film thickness around the tubular pipe. Chen et al. [19] employed the liquid-gas interfacial friction factor equation for wavy annular flow.

According to some experimental studies and field observations, liquid loading begins in deviated wells much earlier than in vertical wells [[17], [18], [19], [20]]. Other researchers have made several attempts to develop models that can aid the calculation of critical gas velocities in gas wells based on two physical theories; the droplet model and film model. It is important to develop a model which shows accurate prediction of critical gas velocity for deviated wells to improve the diagnosis of liquid loading as well as, improve the liquid management strategy for the wells. Thus, the aim of this study is to develop a mathematical model that shows significant modifications and improvements to existing liquid loading models using liquid film flow reversal concept. The new model also has the ability to determine the effect of friction factor and film thickness which directly affect deviated gas wells.

## Methodology

Identification and definition of the exact problem is the first step in proffering the best solution technique to be adopted in predicting liquid loading because, not every solution technique may be appropriate. The concept development simply involves the basis of liquid film flow reversal as the controlling factor for the onset of liquid loading in gas condensate wells. The Model was developed on the basis of minimum kinetic energy criterion focusing on the governing equation used in developing the four-phase model discussed in [4]. The minimum kinetic energy criterion requires that gas kinetic energy must exceed a minimum value so as to be able transport liquid droplets up a gas well. The four-phase annular flow ensures accurate prediction of pressure as well as fluid density for kinetic energy calculations.

There are significant differences in predicting the onset of liquid loading between vertical, directional, and horizontal gas wells. So far, most of the critical models for predicting critical gas velocity in horizontal wells from literature, are based on the droplet model, and the corresponding predictions are far from real conditions and quite contradictory. When liquid loading is present, the gas well experiences a rapid decrease in gas flow and finally stops production. Accurate prediction of the start of liquid loading is crucial for operators to optimize production or take other measures in a timely manner. It was assumed that the flow in the well was one-dimensional. Water drops in the gas core are subject only to gravity, drag and buoyancy forces. The concept of liquid film reversal has been identified as the main concept responsible for generating liquid loading in deviated gas wells. The calculation of the critical gas velocity is considered the most suitable method for determining the beginning of liquid loading.

**Fig. 1:**
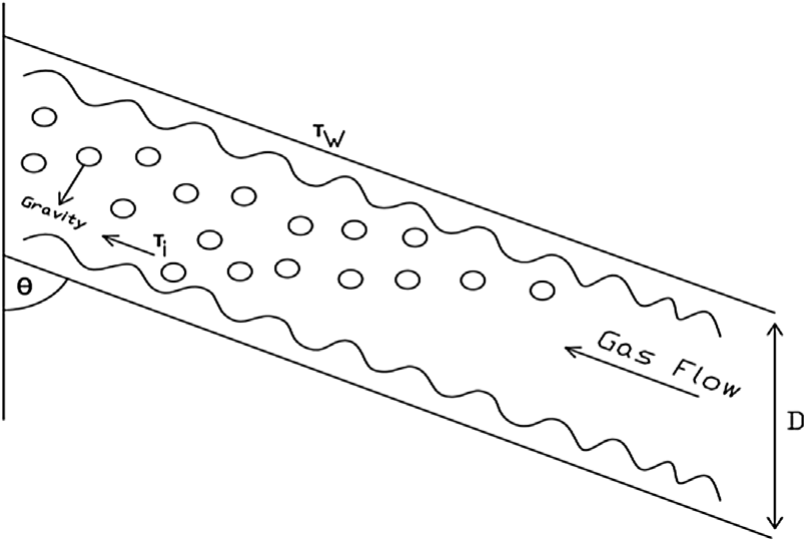
Deviated well liquid film flow model diagram.

Furthermore, field data was used in evaluating the model’s performance. Fig. 1 shows the liquid film flow for the deviated well considered in this study.

### Assumptions

1 The thickness of the liquid film is not uniform around the wall of the pipe
2 The pressure gradient is the same in the liquid and gas phases
3 Effects of gravity and acceleration are not critical for the liquid and gas phases

The steps adopted:

1 Estimation of shear stress
2 Application of the critical film thickness
3 Determination of the critical gas velocity

### Estimation of shear stress

The momentum equation was derived based on the general force balance which is: Flow of fluid momentum into the well section – flow of fluid momentum out of the well section = time rate of change of momentum in the well section.

Force balance for the entire fluid (gas core and liquid film) and can be written as:

(1) $$-\frac{dp}{dx}\frac{\pi D^{2}}{4}+\tau_{w}\pi D-\left[\rho_{l}\left(1-\beta \right)+\rho_{g}\beta \right]\;g\frac{\pi D^{2}}{4}\cos\;\;\theta =0$$

where p = pressure (MPa); x = direction of flow; D = diameter of tubular pipe (m); $\tau_{w}\;$= liquid – wall shear stress (MPa); $\;\rho_{l}$ and $\rho_{g}$ = densities of liquid and gas respectively (kg/m^3^); β = volumetric gas concentration; g = acceleration due to gravity (m/s^2^); θ = deviation angle in degrees.

Force balance for the gas core can be written as:

(2) $$-\frac{dp}{dx}\frac{\pi D^{2}}{4}\beta -\tau_{i}\pi D\sqrt{\beta }-\rho_{g}g\frac{\pi D^{2}}{4}\beta \cos\;\;\theta =0$$

where $\tau_{i}$ = liquid-gas interfacial shear stress (MPa).

Dividing through Eq. (2) by β yields:

(3) $$-\frac{dp}{dx}\frac{\pi D^{2}}{4}-\tau_{i}\frac{\pi D}{\sqrt{\beta }}-\rho_{g}g\frac{\pi D^{2}}{4}\cos\;\;\theta =0$$

Adopting the combined momentum Eqs. (1) and (3) for annular flow pattern gives:

(4) $$\frac{4\tau_{w}}{D}+\frac{4\tau_{i}}{D\sqrt{\beta }}-\;\left(\rho_{l}-\rho_{g}\right)\text{g}\left(1-\beta \right)\cos\;\;\theta =0$$

Considering liquid wall shear stress, liquid interfacial friction factor and the flow coefficients used by Taitler and Dukler [21];

(5) $$\tau_{w}=C_{w}{\left(\frac{DV_{l}\rho_{l}}{\mu_{l}}\right)}^{-a}\frac{\rho_{l}V_{l}^{2}}{2}$$

(6) $$V_{l}=\;\frac{V_{sl}}{\left(1-\beta \right)}$$

Substituting Eq. (5) into Eq. (4);

(7) $$\frac{4C_{w}}{D}{\left(\frac{DV_{sl}\rho_{l}}{\mu_{l}\left(1-\beta \right)}\right)}^{-a}\frac{\rho_{l}V_{sl}^{2}}{2{\left(1-\beta \right)}^{2}}+\frac{4\tau_{i}}{D\sqrt{\beta }}-\;\left(\rho_{l}-\rho_{g}\right)\text{g}\left(1-\beta \right)\cos\;\theta =0$$

Making $\tau_{i}$ subject of formula gives:

(8) $$\tau_{i}=\;\frac{D\sqrt{\beta }}{4}\left(\rho_{l}-\rho_{g}\right)\text{g}\left(1-\beta \right)\;\cos\;\;\theta -\frac{C_{w}\sqrt{\beta }}{2}{\left(\frac{DV_{sl}\rho_{l}}{\mu_{l}\left(1-\beta \right)}\right)}^{-a}\frac{\rho_{l}V_{sl}^{2}}{{\left(1-\beta \right)}^{2}}$$

Where, $\tau_{w}$ = Liquid-wall shear stress, $f_{w}$ = liquid interfacial friction factor and $V_{sl}$ = superficial liquid velocity, m/s^2^.

The evaluation presented by Hewitt & Nicholls [22] reveals that in a two-phase annular flow, the wave height is approximately four to six times of the average film thickness. Droplets are generated by entrainment near a liquid film caused by a gas core. Due to fluctuations in pressure and temperature in the well, the structure of the flow in the well changes from the bottom of the well to the well-head. The diameter of an elongated droplet inevitably affects the size of the droplet in the gas core. Therefore, a more detailed, simple and easy to use model is required. To do this, we examined the method of droplets entrainment in the well, and introduce the effect on the proposed model. Considering the dimensionless film thickness, the liquid void fraction can be expressed as;

(9) $$1-\beta =\;\frac{4\delta }{D}(1-\frac{\delta }{D})$$

and

(10) $$\bar{\delta }=\;\frac{\delta }{D}$$

where δ = film thickness, m; $\bar{\delta }$ = dimensionless film thickness.

Eq. (9) becomes,

(11) $$1-\beta =\;4\bar{\delta }-4{\bar{\delta }}^{2}$$

(12) $$\beta =1-4\bar{\delta }-4{\bar{\delta }}^{2}={\left(1-\;2\bar{\delta }\right)}^{2}$$

The liquid-gas interfacial shear stress, $\tau_{i}$ after substituting Eqs. (11) and (12) in 8 gives:

(13) $$\begin{array}{l} \tau_{i}=\;\left(\rho_{l}-\rho_{g}\right)\text{g}D\;\;\cos\;\theta \left(1-\;2\bar{\delta }\right)\left(\bar{\delta }-{\bar{\delta }}^{2}\right)-\frac{1}{2}C_{w}\rho_{l}{\left(\frac{D\rho_{l}}{\mu_{l}}\right)}^{-a}V_{sl}^{2-a}{\left(\frac{1}{\left(4\bar{\delta }-4{\bar{\delta }}^{2}\right)}\right)}^{2-a}\left(1-\;2\bar{\delta }\right) \end{array}$$

### The critical gas velocity calculation

Chen et al. [19] employed the liquid-gas interfacial friction factor equation proposed by Wallis [22] for wavy annular flow, which is restricted to small film thickness:

(14) $$f_{i}=\;f_{w}\left(1+300\frac{\delta }{D}\right)$$

Fore [24] proposed a liquid gas interfacial friction factor which accounts for large film thickness:

(15) $$f_{i}=0.005\{1+300[(1+\frac{17500}{NRe_{g}}\frac{\delta }{D}-0.0015]\}$$

The modified Luo [18] film thickness correlation was applied to the new model and is given as:

(16) $$\delta \left(\phi ,\theta \right)=(\frac{\theta }{60}\sin(\phi -90)+1)\delta_{c}\;\text{for}\;\;0\leq \theta \leq 60{}^{\circ}$$

(17) $$\delta \left(\phi ,\theta \right)=\;\sin(\phi -90)+1)\delta_{c}\;\text{for}\;\theta >60{}^{\circ}$$

Combining the liquid gas interfacial shear stress given as:

(18) $$\tau_{i}=f_{i}\frac{\rho_{g}V_{sg}^{2}}{2{\left(1-2\bar{\delta }\right)}^{4}}$$

with the compressed liquid-gas interfacial shear stress, $\tau_{i}$ expression in Eq. (19) gives Eq. (20).

(19) $$\tau_{i}=\;\frac{D\sqrt{\beta }}{4}\left(\rho_{l}-\rho_{g}\right)\text{g}\left(1-\beta \right)\;\;\cos\;\;\theta -\tau_{w}\sqrt{\beta }$$

(20) $$0.5f_{i}\rho_{g}V_{g}^{2}\;=\frac{D\sqrt{\beta }}{4}\left(\rho_{l}-\rho_{g}\right)\text{g}\left(1-\beta \right)\;\text{cos}\;\theta -\tau_{w}\sqrt{\beta }$$

Making V_g_ subject of Eq. (20) gives:

(21) $$V_{g}^{2}=\frac{\frac{D\sqrt{\beta }}{4}\left(\rho_{l}-\rho_{g}\right)\text{g}\left(1-\beta \right)\text{cos}\;\theta \;-\;\tau_{w}\sqrt{\beta }}{0.5f_{i}\rho_{g}}$$

(22) $$V_{g}=\sqrt{\frac{D\sqrt{\beta }\left(\rho_{l}-\rho_{g}\right)\text{g}\left(1-\beta \right)\text{cos}\;\theta \;-\;2\tau_{w}\sqrt{\beta }}{0.5f_{i}\rho_{g}}}$$

Applying the Shi [25] assumption on film balance and substituting for β gives the final equation (Eq. (23)) for estimating the gas velocity for the onset of liquid loading in this study.

(23) $$V_{g}=\sqrt{\frac{2g\delta \left(1-3\bar{\delta }+3{\bar{\delta }}^{2}\right)\left(\rho_{l}-\rho_{g}\right)cos\theta }{fi\rho_{g}}}$$

In the early stages, a water-yielding gas well has enough energy for natural flow. During the gas production period, reservoir pressure and gas production rate will gradually decrease, and the liquid-carrying ability will also decrease. If the gas cannot lift the formation water from the wellbore, the fluid will remain in the lower hole, causing a buildup of fluid, called liquid loading. Critical gas velocity is an important control factor to avoid liquid loading. If the gas flow rate is greater than the critical fluid flow rate, the liquid loading will not occur. Therefore, it is important to accurately predict critical gas velocities for sufficient stimulation to avoid liquid loading and increase gas recovery. Thus, the importance of the proposed model (Eq. (22)); liquid loading can seriously damage gas well production.

**Fig. 2:**
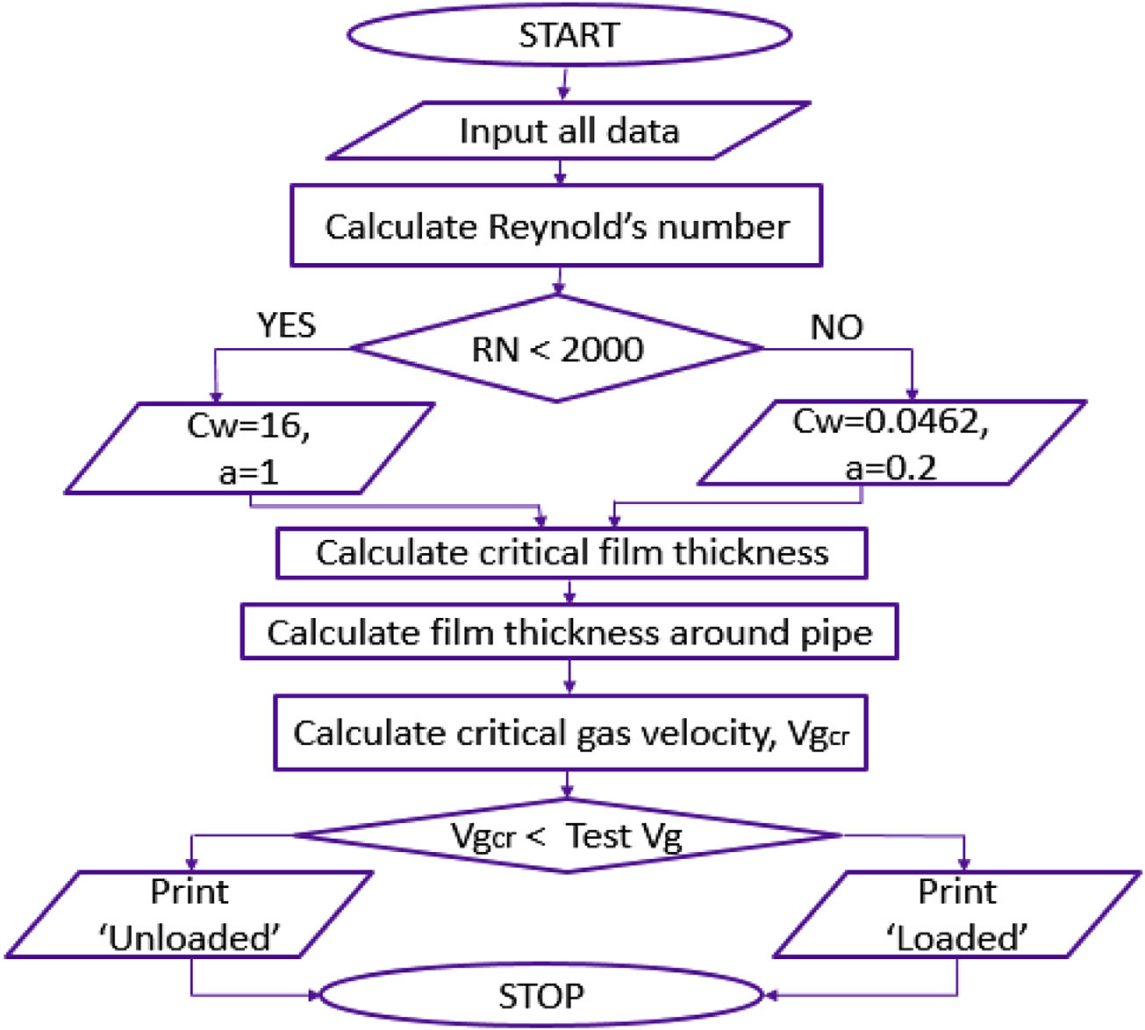
Flowchart showing Model Application steps. *Vg and Vgcr are actual gas and critical gas velocity respectively.

Fig. 2 illustrates the new model flow chart.

## Results and discussion

Gao’s published test data [23] was used to validate the model. The Chuanxi and Daniudi gas fields consist of 42 gas wells, including thirty (30) loaded wells and 12 unloaded wells. For the gas wells, the inclination angle ranged from 20° to 50°, tubing sizes ranged from 2 to 4 in., gas flow rate ranged from 0.49 × 10^4^ m^3^/d to 10.81 × 10^4^ m^3^/d, and liquid flow rate was from 0.03 m^3^/d to 8.0 m^3^/d. Gao’s test data as well as the predicted results using three (3) different models are presented in Table 1, Table 2. Column 8, Column 10 and Column 12 list the predicted status of the new model, Chen’s model and Turner’s model respectively. ***LD*** represents the results predicted as loaded wells i.e. liquid load-up is expected in these wells. ***UNLD*** represents the results predicted as unloaded wells i.e. liquid load-up is not expected in these wells. If the results predicted by the model is the same as the results observed from the well tests, then the prediction is accurate. If the results predicted by the model are contrary to the results obtained from the well tests, then it is said to be incorrect. It should be noted that this model is applicable to gas wells producing water because the predictive correlation of droplet size is only for wells producing water. The predicted critical velocity was compared with the actual gas velocity to determine the liquid loading. If the actual gas velocity is sufficient enough (i.e. higher than the calculated critical gas velocity), there would not be any case of liquid loading in the well, thus the gas well is considered unloaded.

**Table 1:** Data sets from Gao's 30 Loaded wells and predicted results from the current proposed Model and existing Models.

Well No.	gas rate (10^4^ m3/d)	liquid rate (m3/d)	tubing diameter (in)	current gas velocity (m/s)	inclination angle (degree)	Proposed model (critical gas velocity)	Proposed model (predicting status)	Chen model (critical gas velocity)	Chen model (predicting status)	turner model (critical gas velocity)	turner model (predicting status)	Actual status of the wells
1	1.01	0.22	2.875	1.2	35	2.29	LD	1.6	LD	1.8	LD	LD
2	1.16	0.51	2.375	0.31	45	2.37	LD	1.26	LD	1.89	LD	LD
3	0.49	1.23	2.375	0.16	40	1.93	LD	1.7	LD	1.81	LD	LD
4	4.86	0.25	2.375	1.61	24	2.31	LD	2.57	LD	2.77	LD	LD
5	2.93	0.16	2.375	1.29	28	2.21	LD	1.77	LD	1.97	LD	LD
6	2.02	0.51	3.5	0.23	50	1.55	LD	1.2	LD	1.5	LD	LD
7	9.76	0.26	3.5	1.14	40	2.26	LD	1.94	LD	2.24	LD	LD
8	0.99	0.57	3.5	0.36	42	1.78	LD	1.55	LD	2.2	LD	LD
9	0.51	1.23	3.5	0.41	45	1.31	LD	1.54	LD	1.84	LD	LD
10	2.91	0.03	3.5	2.35	50	2.77	LD	3.15	LD	3.85	LD	LD
11	1.39	0.2	3.5	1.12	30	2.67	LD	1.99	LD	2.09	LD	LD
12	0.78	0.06	2.375	1.36	35	2.03	LD	1.98	LD	2.2	LD	LD
13	0.86	0.16	2.441	1.42	32	2.18	LD	1.96	LD	2.2	LD	LD
14	1.08	3.13	2.441	1.78	30	2.24	LD	1.9	LD	2.2	LD	LD
15	0.53	0.57	2.441	0.88	28	2.30	LD	2.23	LD	2.73	LD	LD
16	1.24	0.3	2.441	2.06	30	2.73	LD	2.94	LD	3.64	LD	LD
17	1.63	0.48	2.441	2.7	35	3.91	LD	4.1	LD	4.6	LD	LD
18	3.52	0.36	2.875	4.2	30	4.58	LD	4.48	LD	4.78	LD	LD
19	3.85	0.13	2.875	4.6	25	5.02	LD	5.38	LD	6.18	LD	LD
20	2.68	0.22	2.875	3.2	26	2.99	UNLD	2.18	UNLD	2.58	UNLD	LD
21	2.93	0.51	2.875	3.5	28	3.92	LD	3.98	LD	4.18	LD	LD
22	3.18	0.36	2.875	3.8	35	4.67	LD	4.68	LD	5.28	LD	LD
23	4.22	0.2	3.5	3.4	30	3.67	LD	4.48	LD	4.98	LD	LD
24	5.22	0.4	3.5	4.2	25	4.80	LD	2.8	UNLD	3.1	UNLD	LD
25	4.1	0.3	3.5	3.3	28	3.74	LD	1.78	UNLD	1.98	UNLD	LD
26	4.84	0.57	3.5	3.9	38	5.09	LD	5.28	LD	5.78	LD	LD
27	2	0.43	2.375	3.5	40	4.99	LD	4.58	LD	5.18	LD	LD
28	1.49	1.01	2.375	2.6	50	3.49	LD	3.38	LD	3.68	LD	LD
29	1.66	1.23	2.375	2.9	45	3.98	LD	3.48	LD	4.18	LD	LD
30	1.83	0.25	2.375	3.2	48	3.94	LD	3.58	LD	4.18	LD	LD

*NOTE*: LD = Loading and UNLD = Unloading.

**Table 2:** Data sets from Gao's 12 unloaded wells and predicted results from the new model.

Well No.	gas rate (10^4^ m3/d)	liquid rate (m3/d)	tubing diameter (in)	current gas velocity (m/s)	inclination angle (degree)	Proposed model (critical gas velocity)	Proposed model (predicting status)	Chen model (critical gas velocity)	Chen model (predicting status)	turner model (critical gas velocity)	turner model (predicting status)	Actual status of the wells
1	1.07	0.18	2.375	1.55	20	2.42	LU	2.48	LU	2.78	LU	UNL
2	5.22	0.03	2.441	3.2	30	2.99	UNL	2.88	UNL	3.78	LU	UNL
3	4.21	0.1	2.441	2.2	30	2.18	UNL	1.65	UNL	2.65	LU	UNL
4	5.71	8	2.441	3.8	30	3.53	UNL	3.35	UNL	4.15	LU	UNL
5	2.24	0.17	2.441	2.82	50	1.81	UNL	1.88	UNL	3.28	LU	UNL
6	2.39	0.6	2.375	3.04	20	2.42	UNL	2.49	UNL	3.49	LU	UNL
7	2.65	0.2	2.375	2.12	45	1.86	UNL	1.61	UNL	2.15	LU	UNL
8	7.46	0.14	2.375	3.52	25	2.29	UNL	2.75	UNL	3.95	LU	UNL
9	3.43	0.5	2.375	3.6	22	3.24	UNL	3.08	UNL	4.28	LU	UNL
10	3.9	0.2	2.875	2.5	26	2.19	UNL	2.18	UNL	3.38	LU	UNL
11	10.81	0.16	2.875	4.2	30	3.85	UNL	3.58	UNL	4.98	LU	UNL
12	5.67	0.1	2.875	5.2	32	2.78	UNL	2.68	UNL	2.98	UNL	UNL

*NOTE:* LU = loading and UNL = Unloading.

In Table 1, it is clearly obvious that the new model performed better than the Chen et al model owing to the fact that, at a liquid flow rate of ≥0.3, the Chen’s model fails the test as it wrongly depicts the status of the well; these results were actually seen for well-angular inclinations of 25, 28 and 30°. Based on the results also obtained in Table 2, it was observed that, the new model will behave like the Chen’s model when the liquid flow rate is less 0.3; because it is observed that liquid film flow reversal is a controlling factor for the onset of liquid loading in this cases. However, despite increasing the angular inclination of the well, the new model’s accuracy will not drop for even lower liquid flow rates of 0.03 m^3^/d because, it was discovered that, at such condition, so long as the gas flow rate is in the region of 4.1-5.55 m^3^/d, the new model still retains its accuracy.

**Fig. 3:**
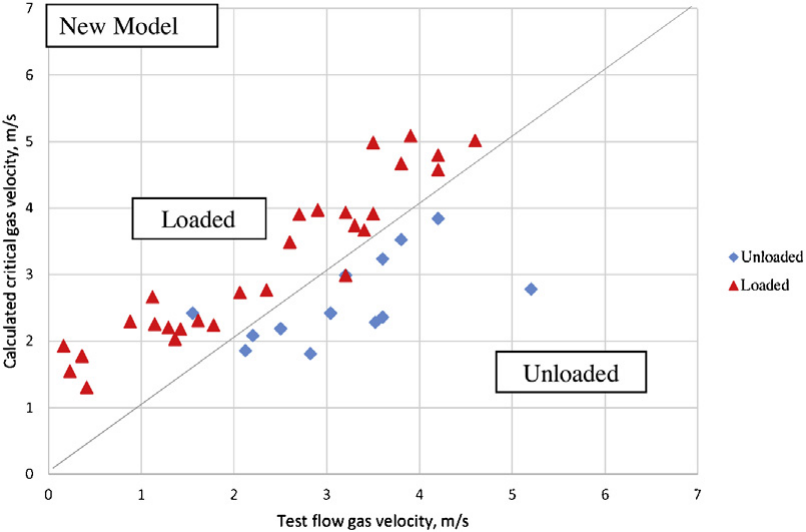
Proposed Model-Calculated Critical Gas Velocity Vs. Test Flow Gas Velocity.

**Fig. 4:**
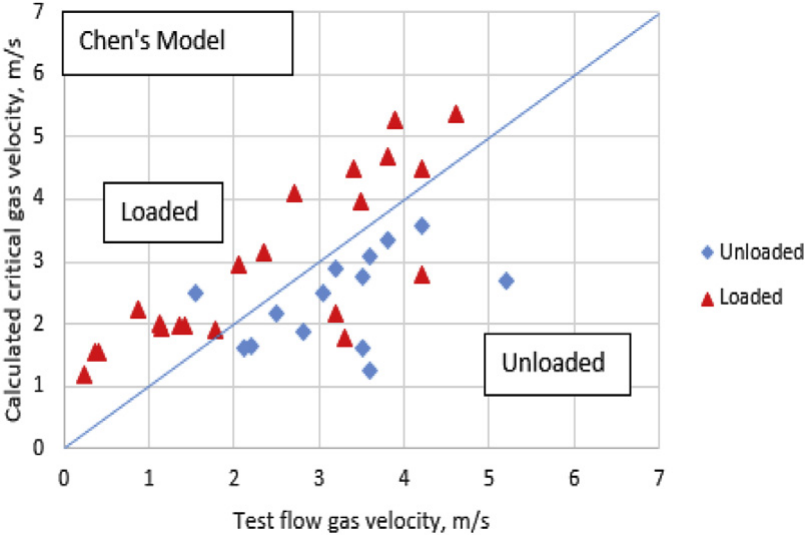
Chen’s Model-Calculated Critical Gas Velocity Vs. Test Flow Gas Velocity.

In Fig. 3, the proposed modified Chen et al. [19] model predictions for the loaded wells as determined in Gao’s well test data are identified with red triangles. Unloaded wells are identified with blue diamonds. A forty-five degrees (45°) line was used to divide the figure into two sections. The upper region is where the loaded wells should reside while the lower section is for the unloaded wells. The proposed model shows a much better prediction of liquid loading in the gas wells when compared with Chen’s model prediction in Fig. 4, with the data from 11 out of 12 unloaded wells correctly fitting the new-model predictions; and the data accuracy of 29 out of 30 loaded wells with those of the new model also show the level of accuracy exhibited by the new model. Thus, the new model is a better model for predicting liquid loading in deviated gas wells.

As the gas-liquid ratio increases with increase in gas production rate, the risk of liquid reduces thus affecting the hole/tube diameter. The tube diameter significantly affects the gas mass flow rate. The dimensionless critical gas mass flow rate increases with an increase in the tube diameter. The inclination angle has a significant impact on the film distribution in the circumferential position of the pipe, and this is evidenced in the predictions made with the proposed and existing models (Table 1). In horizontal gas wells, the liquid film in the upper part of the gas core is thinner than that in the lower part, which is dominated by gravity effects. As the well deviates from being vertical, there is an increase in the film thickness located at the bottom of the pipe. Considering its simple form for calculating the critical gas velocity in inclined pipes, it can be claimed that the new model can provide gas production engineers with a convenient and accurate approach to predicting the critical gas velocity. This method has brought to light a means of predicting liquid loading in deviated natural gas wells by employing the concept of liquid film flow reversal. The concept of non-uniform film thickness incorporated in developing the new model, as well as a modified friction factor have actually helped to improve the performance of the model. Hence, these two modifications have brought about an improved model for determining gas well liquid-load up in deviated gas wells.

## Conclusion

This study is geared towards determining liquid load-up in deviated gas wells by modifying friction factor and accounting for non-uniform film thickness in the developed model. In lieu of the several attempts made by other researchers to develop models that can aid the calculation of critical gas velocity in gas wells based on two physical theories (droplet model and film model), this study is an improvement on the liquid loading model developed by Chen et al. The new gas well liquid loading model was developed with a focus on two key issues i.e. the consideration for film thickness and the inclusion of a different interfacial friction factor. The model has the ability to predict the onset of liquid loading in deviated gas wells. The model introduces the concept of non-uniform film thickness around the pipe well, as against previous studies which considered uniform film thickness.
